# Enhancement of Skin Barrier Function Using DYNAMiQ Technology: A Clinical Evaluation

**DOI:** 10.1111/jocd.70889

**Published:** 2026-05-05

**Authors:** Klaus Fritz, Mariya Genova, Carmen Salavastru

**Affiliations:** ^1^ Dermatology and Laser Center Landau in der Pfalz Germany; ^2^ Individual Practice for Specialized Medical Dermatology Care Mariya Genova MD Plovdiv BG Bulgaria; ^3^ Carol Davila University Bucharest Romania; ^4^ Department of Dermatology Colentina Clinical Hospital Bucharest Romania

**Keywords:** calcium gradient, hydration, skin barrier, skin health, TEWL

## Abstract

**Background:**

The integrity of the skin barrier is fundamental to maintaining overall skin health, serving as the primary defense against environmental insults. Disruption of this barrier is linked to various dermatological issues, including inflammation, sensitivity, and premature aging. Therefore, technologies designed to reinforce the skin barrier and enhance moisture retention hold a significant promise for improving skin condition, resilience, and overall function.

**Aims:**

To evaluate the restorative effect of DYNAMiQ technology on skin barrier function.

**Methods:**

This prospective, single‐center study enrolled 24 female subjects (*n* = 24, 26–64 years). Each subject received one DYNAMiQ treatment, with a follow‐up assessment performed 7 days later. Transepidermal water loss (TEWL) was measured before treatment, immediately after, and at follow‐up. Subject‐reported outcomes were collected using comfort and satisfaction questionnaires, and standardized photographs were taken.

**Results:**

TEWL increased slightly from a baseline of 11.95 ± 0.24 g/m^2^/h to 12.70 ± 0.19 g/m^2^/h immediately post‐treatment. By day 7, TEWL decreased significantly to 6.63 ± 0.18 g/m^2^/h, representing an 80.3% improvement in water vapor loss. All subjects (100%) expressed satisfaction with the results, and 96% reported that their skin felt more hydrated and nourished.

**Conclusions:**

DYNAMiQ technology effectively improves skin barrier function by significantly reducing TEWL. These findings are supported by high subject satisfaction and perceived hydration improvements.

## 1 Introduction

Human skin plays several vital roles, including protection, sensory perception, and maintenance of homeostasis. Central to these functions is the skin barrier, which acts as a dynamic interface between the body and the external environment [1]. It provides defense against physical, chemical, and microbial threats. In addition to its biological importance, the condition of the skin barrier also influences the aesthetic appearance of the skin, including surface texture, tone, and elasticity [2].

Skin aging is a complex biological process that progressively alters both epidermal and dermal compartments [3]. A key characteristic of aged skin is the deterioration of the barrier's structural integrity. With age, the epidermal layer becomes thinner due to keratinocyte atrophy, and the skin's resilience to external insult declines [4]. Compromised structural proteins increase susceptibility to microbial infiltration and slow recovery following barrier disruption [5, 6]. These changes initiate a self‐reinforcing cycle in which barrier weakening promotes inflammation, accelerating further aging‐related downturn [7]. Although symptomatic treatments exist, strategies that directly address the root cause of barrier dysfunction and inflammation remain limited.

Among the emerging therapeutic strategies, restoration of the epidermal calcium gradient has received growing attention due to its essential role in barrier formation and repair. This calcium gradient, rising from the basal layer to the stratum granulosum, is crucial for regulating keratinocyte differentiation and lipid processing during the formation of the stratum corneum (SC) [8, 9]. Disruption of this gradient with age or external stress diminishes skin barrier homeostasis.

To address this fundamental disruption, DYNAMiQ technology has been developed to restore the calcium gradient within the epidermis. It utilizes a sonic transducer to convert electrical energy into low‐frequency mechanical oscillations. When applied to the skin, these sonic waves induce cellular oscillations that stimulate calcium ion influx and propagate extracellular calcium waves across the epidermis [10, 11, 12]. Repeated stimulation aims to reestablish the calcium gradient, thereby supporting natural regenerative processes and improving the skin's barrier function.

While hydration and transepidermal water loss (TEWL) are downstream indicators of barrier status, their clinical relevance lies in their ability to non‐invasively reflect functional integrity of the skin. The SC, the outermost layer of the epidermis, regulates water retention through a highly organized structure of corneocytes (“the bricks”) and intercellular lipids (“the mortar”) [13]. This structure provides mechanical strength and hydrophobic resistance to water loss. Disruption of the SC, particularly its lipid matrix composed of ceramides, cholesterol, and free fatty acids, leads to elevated TEWL and barrier compromise [14, 15].

TEWL, defined as the passive diffusion of water from the viable epidermis through the SC, is widely used as a quantitative marker of barrier function [16, 17, 18]. Under healthy conditions, TEWL remains low, reflecting an intact, well‐organized barrier. Elevations in TEWL indicate impaired water retention capacity and are commonly observed in dermatologic disorders such as atopic dermatitis and psoriasis [16, 19, 20, 21, 22, 23].

This study aims to evaluate the effects of DYNAMiQ technology on the structural and functional integrity of the skin barrier, using TEWL as a non‐invasive, quantitative biomarker of barrier status.

## 2 Methods

In this prospective, single‐center study, 24 female subjects (*n* = 24, 26–64 years) were enrolled to evaluate the safety and performance of the EMFUSION device (BTL Industries Inc., Boston, MA), intended to improve the function of the skin barrier. Epidermal permeability barrier function was assessed using TEWL measurements before treatment, after treatment, and at follow‐up, accompanied by photographic documentation of the face at baseline and at follow‐up. In addition to objective assessments, subject‐reported outcomes related to treatment comfort and satisfaction were collected using standardized questionnaires. Informed consent was obtained from all participants, and adverse events were monitored throughout the study.

Inclusion criteria required participants to have facial skin prone to dryness and to be willing to comply with study procedures and scheduling. Exclusion criteria included active acne, infections or open wounds, autoimmune or communicable disease, cold sores or fever blisters, and any other concurrent facial treatments that may interfere with the study therapy.

### 2.1 Treatment Protocol

Subjects underwent a single 25‐min EMFUSION procedure, with a follow‐up visit conducted 7 days post‐treatment.

Before the session, the treatment room was monitored for temperature fluctuations to ensure that environmental conditions (e.g., ambient temperature and humidity) did not interfere with the procedure. Therapy settings were adjusted based on individual subject feedback to maximize comfort and effectiveness. After the treatment, participants were instructed to apply the Sealing Gel once daily for several days as part of the standard post‐treatment protocol. The gel is intended to support short‐term skin recovery following the procedure rather than to provide a long‐term therapeutic effect on skin barrier function. To minimize the potential influence of transient occlusive or humectant effects on barrier assessment, subjects discontinued use of the Sealing Gel at least 48 h before the final TEWL measurement. This washout period was selected to reduce the likelihood that endpoint measurements were affected by short‐term topical effects.

### 2.2 TEWL Measurement

TEWL was assessed using the Multi Skin Test Center MC 750 equipped with a Tewameter probe (Courage + Khazaka electronic GmbH, Köln, GE) at three time points: baseline, immediately after treatment, and seven days post‐treatment. The device calculated the rate of water vapor loss (in g/m^2^/h) by measuring humidity gradients. Before each measurement, subjects rested for 20 min in a room with controlled environmental conditions, with the temperature maintained at 21°C ± 0.8°C and relative humidity between 50% and 55%. Measurements were performed at four facial sites: the left and right sides of the forehead and the left and right cheeks.

### 2.3 Data Collection

Standardized high‐resolution 2D photographs were taken at baseline and follow‐up visit. Photographs were taken from a front angle, with a consistent background and uniform lighting. The Therapy Comfort Questionnaire (TCQ) was completed immediately after treatment and combined a 5‐point Likert Scale with a 10‐point Visual Analog Scale (VAS) for pain assessment, where 0 indicated no pain and 10 represented maximum bearable pain. The Subject Satisfaction Questionnaire (SSQ) was completed at the follow‐up visit and evaluated using the 5‐point Likert Scale.

### 2.4 Statistical Analysis

Statistical analysis was conducted using GraphPad Prism software. Descriptive statistics, including mean and standard deviation, were calculated. The non‐parametric Friedman test was used to assess the statistical significance of changes in TEWL measurements over time. A significance level of α = 0.05 was adopted.

## 3 Results

A total of 24 subjects (*n* = 24, females) participated in this study. All subjects completed the treatment session and follow‐up visit. No adverse events were observed, and no one was withdrawn throughout the study.

### 3.1 TEWL Measurement

**FIGURE 1:**
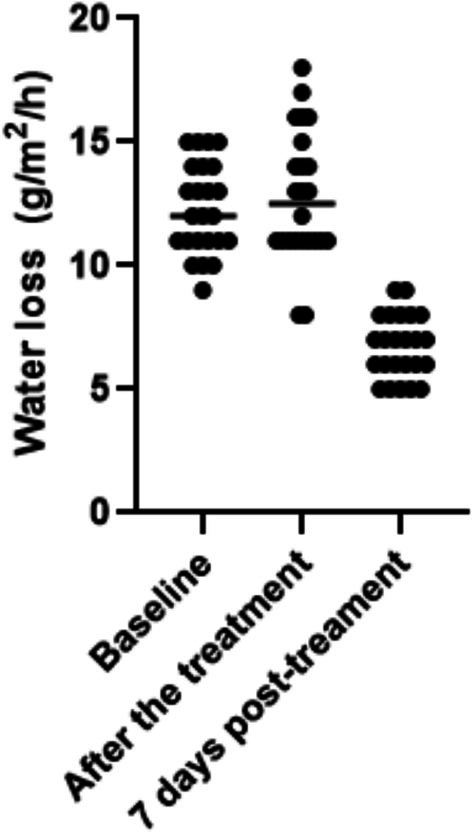
The amount of water evaporated through the skin on the left cheek, measured by TEWL (g/m^2^/h), was assessed at multiple time points. Before treatment, subjects showed elevated TEWL levels, which increased slightly immediately after treatment, suggesting a temporary disruption of the skin barrier. By seven days post‐treatment, TEWL had significantly decreased, indicating a marked improvement in barrier function.

TEWL was successfully measured in all subjects. The measurement showed significant improvements (*p* < 0.0001) in the amount of water passively evaporating through the skin. The average baseline value was 11.95 ± 0.24 g/m^2^/h. Immediately after the treatment, the value increased to 12.70 ± 0.19 g/m^2^/h. Seven days post‐treatment was observed a notable decrease was observed to 6.63 ± 0.18 g/m^2^/h, representing an 80.3% improvement in water vapor loss (see Figure 1).

### 3.2 Therapy Comfort and Subject Satisfaction

All subjects strongly agreed with the statement “I found the treatment comfortable” as recorded in the TCQ. In terms of pain assessment, 96% of subjects rated the procedure as 0, indicating “no pain”, while only one subject rated the pain level as 1. In the SSQ, 100% of subjects reported satisfaction with the results. Furthermore, 96% of subjects indicated that their skin felt more hydrated and nourished, noted a reduction in pore size, and observed an overall improvement in the appearance of their skin following the treatment.

## 4 Discussion

The results of this study demonstrate a significant improvement in skin barrier integrity following a single DYNAMiQ treatment session, as reflected by a marked reduction in TEWL. TEWL was used as a non‐invasive, quantitative marker of barrier function, given its sensitivity to changes in epidermal water permeability. Baseline TEWL values averaged 11.95 ± 0.24 g/m^2^/h, decreasing to 6.63 ± 0.18 g/m^2^/h by day 7 post‐treatment, an improvement of 80.3%, indicating substantial reinforcement of the barrier's capacity to retain moisture and resist external stressors.

A slight increase in TEWL was observed immediately after the procedure, which aligns with the mechanism of action of DYNAMiQ technology. The technology generates sonic waves that induce an influx of calcium ions and a dynamic propagation of calcium waves through the epidermis [12]. This process induces a transient disruption of epidermal homeostasis, which is hypothesized to activate regenerative signaling pathways [24]. As a result, immediately post‐treatment, the skin barrier is undergoing acute stimulation and active remodeling, rendering it transiently more permeable and not yet fully stabilized. This controlled disruption initiates the regeneration process, leading to the re‐establishment of skin barrier integrity and a marked improvement in water retention by day 7.

A comparable pattern of transient post‐procedural TEWL elevation followed by sustained barrier recovery has been reported for other energy‐based modalities, including fractional laser and radiofrequency devices, though the increase observed here is notably modest, consistent with the non‐ablative, non‐thermal nature of sonic wave stimulation [25, 26]. By analogy with these biostimulatory modalities, where repeated treatment sessions are generally required to accumulate and sustain structural remodeling, it is plausible that multiple DYNAMiQ sessions may similarly be needed to achieve durable barrier reinforcement beyond the single‐session, seven‐day window evaluated here [27].

A significant decrease in epidermal water evaporation observed at the follow‐up visit confirms the positive restorative effect of DYNAMiQ technology on skin barrier function. Restoration of the barrier integrity is crucial, as it directly correlates with improved skin hydration, reduced sensitivity to external aggressors, and enhanced overall skin health [28]. From a clinical perspective, reinforced barrier function may reduce susceptibility to dehydration, irritation, and external stressors associated with skin dysfunction [29]. These findings were further supported by subject‐reported outcomes, where participants noted visible improvements, including a decrease in pore size and a feeling of nourished, hydrated skin. Perception of smoother texture and smaller pores might reflect improved corneocyte cohesion and surface reflectivity, both of which result from restored SC architecture. Together, these objective and subjective outcomes suggest that DYNAMiQ technology facilitates barrier recovery and may contribute to improved skin resilience, offering a promising approach for preventative and corrective skin treatments.

This experiment provides novel insight into the role of calcium gradient regulation in maintaining skin barrier function. It is proposed that exposure to sonic waves may help restore the calcium gradient, a key marker of healthy skin. The integrity of the epidermis critically depends on calcium ion distribution, with the calcium gradient playing a fundamental role in regulating keratinocyte differentiation, lipid organization, and regulation of epidermal permeability [30]. Understanding and targeting the regulation of the epidermal calcium gradient present significant potential to advance therapeutic strategies for skin barrier restoration. As hydration and water retention are key determinants of skin health, and their disruption is central to a wide range of barrier dysfunctions, strategies that enhance water content and optimize the skin's protective function represent a major step forward in addressing both clinical and aesthetic skin concerns.

Bubley et al. [31]. reported that trichloroacetic acid chemical peeling improved palmoplantar psoriasis by restoring normal skin acidity, enhancing penetration of topical therapies, and supporting barrier repair. While effective, this approach involves controlled injury to the skin. In contrast, DYNAMiQ offers a non‐invasive method to restore calcium balance, potentially activating similar repair pathways and promoting healthy differentiation without the need for chemical or ablative procedures.

In contrast to topical formulations, which rely on passive diffusion and are often constrained to superficial skin layers, DYNAMiQ uses mechanical energy to initiate biological repair processes within the epidermis. By engaging cellular mechanisms involved in barrier restoration, this approach may produce effects that are both deeper and more sustained. The limited penetration of topical agents through the SC is a well‐recognized challenge, particularly when therapeutic action is required in deeper dermal layers. Energy‐based approaches, such as DYNAMiQ, may help address this limitation by modulating cellular activity beyond the reach of conventional topical treatments.

The generalizability of the results is limited by the absence of male participants. Additionally, the study evaluated the effects of only a single procedure, with only one follow‐up visit conducted at seven days post‐treatment. Nevertheless, among the study's strengths is the sufficient sample size of 24 subjects, which provides a solid foundation for statistical analysis and supports the reliability of the observed effects. Moreover, the study was conducted under rigorously controlled conditions, minimizing potential external influences on the measurements. In addition to objective TEWL assessment, subject evaluations regarding comfort, satisfaction with the results, and standardized pre‐ and post‐treatment photographs added valuable depth to the outcome analysis. Further studies would benefit from incorporating multiple treatment sessions, longer follow‐up periods to confirm the durability of the results, and a broader participant population, including men and individuals with higher Fitzpatrick skin types. Such expectations would help characterize the long‐term efficacy and broader applicability of DYNAMiQ technology across diverse populations.

## 5 Conclusion

The results presented in this study demonstrate that DYNAMiQ technology is highly effective in restoring skin barrier function, resulting in a significant reduction in transepidermal water loss. These objective findings are further supported by subject‐reported outcomes, with 96% of subjects noting an improvement in the overall appearance of their skin and 100% expressing satisfaction with the treatment results. DYNAMiQ technology can be considered a safe, effective, and comfortable procedure for enhancing skin barrier integrity.

## Author Contributions

All authors have read and approved the final manuscript.

## Conflicts of Interest

All authors act as clinical investigators for BTL. However, no funding or financial support was received for the research, authorship, or publication of this article.

## Data Availability

The data that support the findings of this study are available on request from the corresponding author. The data are not publicly available due to privacy or ethical restrictions.
